# A simple technique to improve docking time in robotic surgery

**DOI:** 10.1007/s11701-024-02179-z

**Published:** 2024-12-01

**Authors:** Yoav Mintz, Ram Elazary, Brigitte Helou, Ronit Brodie, Gad Marom

**Affiliations:** 1 Department of General Surgery, Hadassah Hebrew University Medical Center, Jerusalem, Israel; 2 Faculty of Medicine, Hebrew University of Jerusalem, Jerusalem, Israel

**Keywords:** Docking, Robotic surgery, Technique, Hugo RAS™, daVinci

## Abstract

The docking process is a crucial step in robotic surgery, which determines the technical capability of moving the robotic arms during surgery and affects the performance of surgery. Robotic companies, therefore, provide setup guides recommending the trocar placement, arm carts, and operating table positioning for each procedure or body quadrant, to facilitate this process. This process takes time and although improves with experience, reported docking times range from 4 to 29 min. The final step of docking the robot is sometimes challenging and includes the insertion of the robotic instruments into the surgical field which involve patient safety. We implemented a simple new technique for safe insertion of robotic instruments and overcome the challenge of manipulating the camera arm for visualizing the trocars and advancing of the robotic instruments into the surgical field. This technique utilizes simple laparoscopic skills of laparoscopic instrument insertion. Docking times were recorded in ten consecutive patients undergoing robotic surgeries with the Hugo RAS™ and compared to our prospectively collected data registry prior to this technique. The new technique reduced the average docking time by 3.5 min and resulted in improvement of 45% in docking time. Integrating trivial laparoscopic skills into the docking process may simplify and reduce the docking time in robotic surgery.

**Supplementary Information:**

The online version contains supplementary material available at 10.1007/s11701-024-02179-z.

## Background

Docking the robotic platform onto the trocars and insertion of the robotic instruments into the surgical cavity is a significant step in robotic surgery, and when done correctly can facilitate the flow of surgery. This is emphasized when contemplating if to undock and redock during surgery, either partially (one arm) or completely.

There are four steps during the docking process:

1. Trocar insertion and positioning the operating room table in the necessary tilt and roll position;
2. Driving the arm cart/s to proximity of the patient;
3. Connecting the arms to the trocars;
4. Insertion of the instruments into the operating field.

While steps 1–3 improve significantly with experience, insertion of the instruments is still a time-consuming step since it involves the safety of patients, demanding special attention and maneuvers. The definition of docking time varies greatly between studies resulting in a wide range of time from an average of 4 to 29 min (1–10). Some define docking time as the time from moving the arm cart until all instruments are inserted and some from skin incision to all instruments inserted. In studies reporting docking time less than 5 min, the docking time is usually defined from moving the arm cart until all arms are connected to the trocars, not including instrument insertion, although this step sometimes takes longer than all steps together.

As opposed to standard laparoscopic surgery, insertion of robotic instruments cannot be performed blindly, since there is no sense of touch or resistance when the instruments are driven into the surgical field using large mechanical arms, which reduce forces by dedicated springs and motors. In addition, there is a tendency to begin insertion of the instruments perpendicular to the skin, and then change the direction of insertion into an acute angle, pointing the instrument to the target zone. Inserting a robotic instrument perpendicular may result in organ injury without necessarily being aware of the injury. This is why the robotic companies insist on inserting the instruments only under direct visualization. Once the camera arm is docked, it is manipulated to visualize the trocar, and only then under direct visualization the instrument should be inserted and advanced to the target zone. This is not always easy since the camera arm needs to be moved significantly to show the abdominal wall and can potentially collide with an adjacent arm already in place. Also visualizing a trocar in close proximity or one that is behind the falciform ligament is particularly challenging. Since locating the trocars for instrument insertion is not trivial, some robotic platforms, like the daVinci Xi, aid the surgeon by presenting their approximate location on the periphery of the screen, thereby moving the camera toward the specific trocar indicator should eventually bring the trocar into view.

Herein, we present a simple technique for safe insertion of robotic instruments, which avoids the necessity of camera manipulation and significantly reduces the overall docking time.

## Methods

**Fig. 1:**
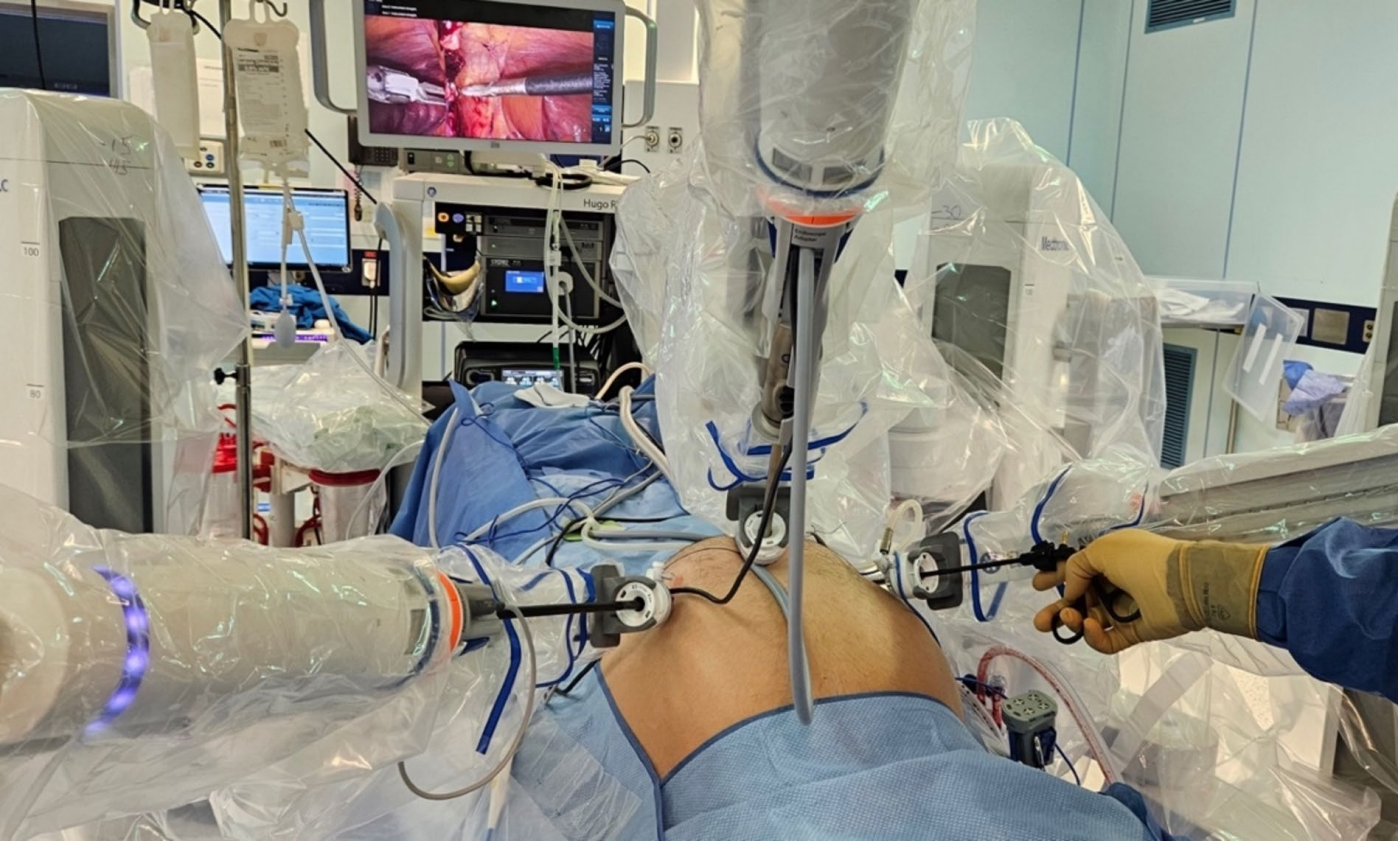
The docking technique demonstrated on the Hugo RAS^TM^ during an inguinal hernia repair. The Camera arm is docked and placed to show the center of the operating field, the left arm is docked and a robotic bipolar fenestrated grasper is in place. A laparoscopic instrument is inserted through the trocar instead of a robotic instrument and is viewed on the screen as it enters the operative field. Once the robotic arm is in the desired position, a robotic instrument may be inserted without visualizing the trocar (A short video in the supplemental material demonstrates the technique)

Docking technique — the docking process steps 1–3 are carried out as usual. Following trocar insertion, the arm carts are wheeled toward the patient’s side and connected to the trocars. Once the camera arm is connected, the camera is inserted and positioned to view the target surgical field. Each instrument arm is then moved so it will position its trocar in an angle pointing directly into the surgical field. Then a standard laparoscopic grasper is inserted through the trocar into the surgical field, while it is still connected to the robotic arm (Fig. 1). If the trocar was positioned adequately, once the instrument is inserted sufficiently it will be visualized in the camera view. Fine adjustments of the tip of the laparoscopic grasper can be made under direct vision by moving the robotic arm accordingly, while holding the grasper in one hand and moving the robotic arm in the other. If the trocar was not positioned adequately, insertion of the laparoscopic grasper will allow the feeling of resistance in case of organ obstruction and will enable adjusting the positioning of the trocar to bring the tip of the grasper to the desired location. Once the robotic arm is repositioned adequately, the laparoscopic grasper is removed and the robotic instrument is inserted until visualized in the surgical field. This technique is repeated for each robotic instrument arm, without requiring movement of the camera arm or locating the trocars.

Although this technique is applicable for any robotic system, the evaluation of this technique was performed only on operations using the Hugo RAS™ system due to an IRB in place. This study was performed in line with the principles of the Declaration of Helsinki. Approval was granted by the Ethics Committee of the Hadassah Hebrew University Medical Center (HMO- 0480–22). Informed consent was obtained from all individual participants included in the study.

## Results

A total of 138 operations were performed in our institution with the Hugo RAS™ system. Data was available for 101 patients, all of whom signed an informed consent to be included in the registry. Operations included inguinal hernia, ventral hernia, cholecystectomy, hiatal hernia repair, Heller myotomy, Nissen fundoplication, gastric bypass, esophagectomy and colectomy. Docking time varied slightly between types of operations but had an average of 7.8 min in the last 40 cases before implementation of the new docking technique. Docking time was defined as beginning from moving the first arm cart, and ending after all robotic instruments were inserted and ready for control by the console surgeon. No distinction was made, at the time, between total docking time and instrument insertion time.

Following implementation of the new technique, docking times were recorded including the specific portion for instrument insertion times in ten consecutive patients. Instrument insertion time was defined from the time all robotic arms were connected to the trocars until all instruments were inserted and ready for surgeon console control. This included the positioning of the arms, insertion of the laparoscopic graspers and insertion of the robotic instruments.

The average total docking time was 4.3 min including instrument insertion (Table 1). Average instrument insertion time was 1.3 min. The total average docking time decreased from 7.8 to 4.3 min, meaning a reduction of 3.5 min in average, and a 45% improvement in docking time.

**Table 1:** Ten consecutive operations with the Hugo RAS^TM^ and their docking times. The instrument insertion time lasts an average of 1.3 min with an average total docking time of 4.3 min

	Operation	Start	Arms connected	Docking time	Instrument ready	Instrument insertion time	Total docking time
1	Esophagectomy	9:48	9:54	6	9:55	1	7
2	Inguinal hernia	18:04	18:07	3	18:09	2	5
3	Inguinal hernia	15:54	15:57	3	15:59	2	5
4	Cholecystectomy	8:53	8:55	2	8:56	1	3
5	Inguinal hernia	11:01	11:03	2	11:04	1	3
6	Inguinal hernia	13:38	13:41	3	13:42	1	4
7	Heller myotomy	16:23	16:26	3	16:28	2	5
8	Inguinal hernia	16:13	16:16	3	16:16	1	4
9	Cholecystectomy	18:16	18:19	3	18:20	1	4
10	Inguinal hernia	9:08	9:10	2	9:10	1	3
	Average time			3		1.3	4.3

## Discussion

Docking of the robotic systems is still a significant issue in robotic surgery. It takes time, it influences the mechanical performance and capabilities of instruments, and involves safety issues. Reported docking times with various systems take no less than 4 min without instrument insertion and up to 29 min from skin incision to all instruments in. Inserting the instruments is the most challenging step comprising a significant proportion of the total docking time. We herein present a new technique for instrument insertion, which maintain safety and reduces docking time by approximately by 45%. Although the instructions for use of the robotic systems dictate the way the docking should be done, we believe that using this technique significantly improves the docking time while maintaining the safety requirements. It grants a sense of control and facilitates the decision to undock and redock during the procedure if necessary. Surgeons must be aware however, that in cases that the direct path from the trocar to the surgical field is obstructed by adhesions, this should be taken care of laparoscopically prior to docking, as this is necessary for instrument exchange during surgery as well. Limitations to our proposed new technique are the low number of cases studied, and that six out of ten cases were inguinal hernia operations, performed with three arms only. Also, the laparoscopic instrument insertion by itself needs laparoscopic expertise. A larger group of patients will be studied to further validate this technique which will include more operations with 4 arms, and we recommend that this technique should be done by experienced laparoscopic surgeons rather than nurses or residents in their initial training.

## Conclusion

Simple laparoscopic skills such as laparoscopic instrument insertion into the surgical field, to accurately position the robotic arms may be integrated into the robotic docking process. This may replace the need for visualizing the trocars during instrument insertion, simplify the docking process and may reduce the overall docking time.

## Supplementary Information

Below is the link to the electronic supplementary material.

Supplementary file1 (MP4 40801 KB)

## Data Availability

Data is provided within the manuscript.
